# Supplementary material for: Observation of elastic topological states in soft materials
Source: Nat Commun. 2018 Apr 10;9:1370. doi: 10.1038/s41467-018-03830-8 (PMC5893582; doi:10.1038/s41467-018-03830-8)
Supplement: Supplementary file 1 — Supplementary Information [file 41467_2018_3830_MOESM1_ESM.pdf]

Supplementary Information

Observation of elastic topological states in soft materials, Li et al.

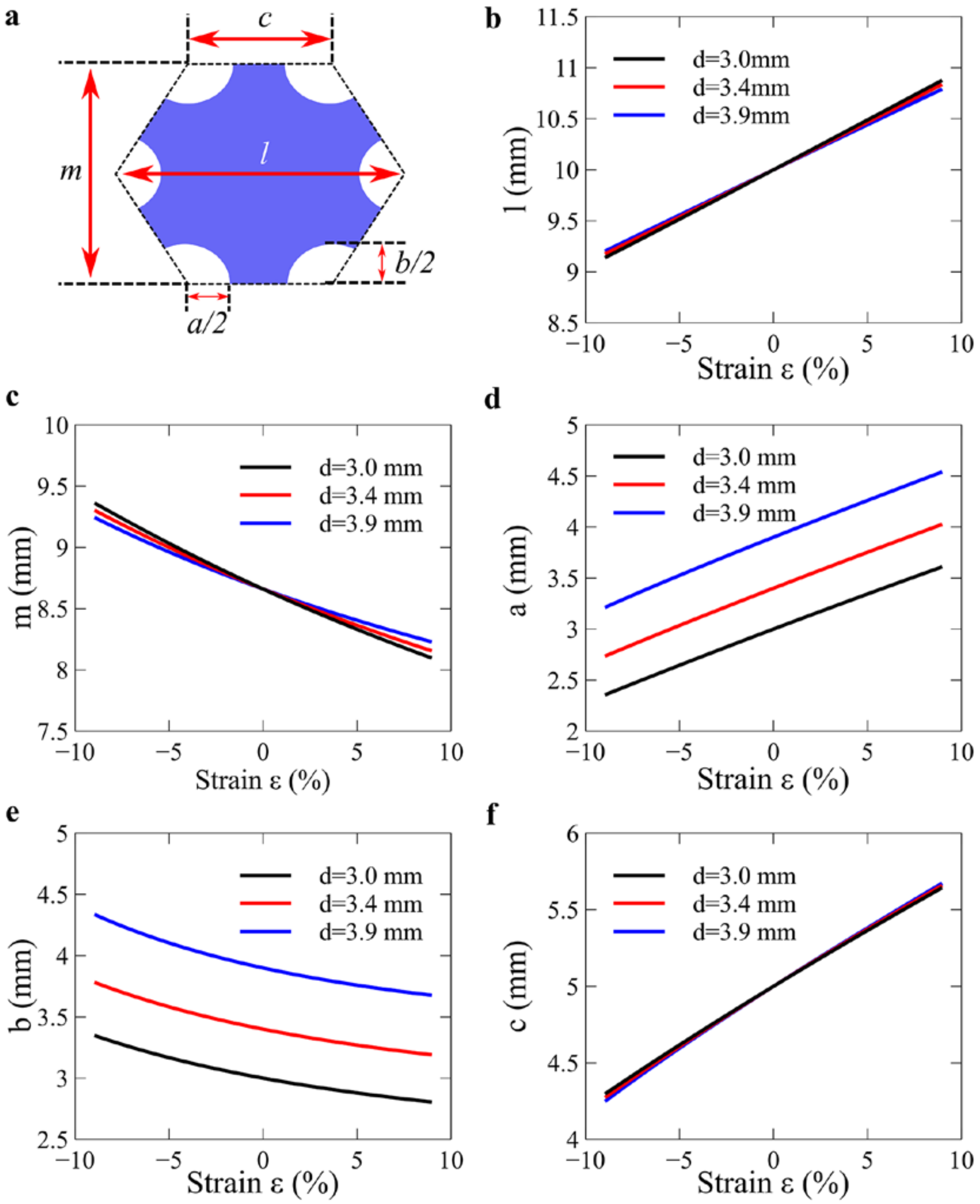

Supplementary Figure 1 | Simulation of shape change of unit cell under uniaxial deformation. **a**, Geometry of unit cell and five parameters determining the geometry of unit cell. **b-f**, The length and width of hexagon cluster, two axes of ellipse and the distance of two holes change as a function of strain.

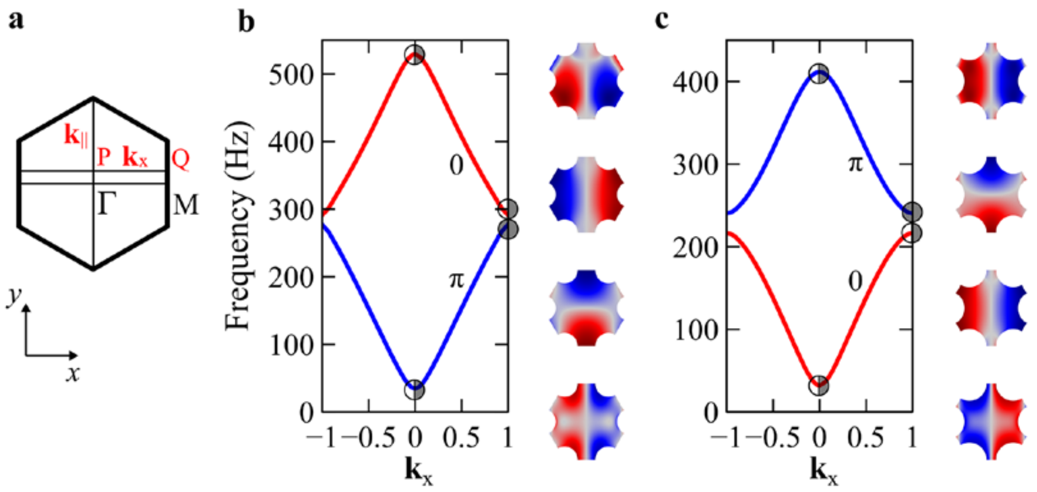

Supplementary Figure 2 | Analysis of mirror symmetry for Zak phase calculation. a, The schematic of Brillouin zone (BZ) with a full $\mathbf{k}_x$ and a fixed $\mathbf{k}_{||} = 0.2\times y_k$. P and Q points denote the center and edge of Brillouin zone. **b-c**, The band structure of d/R = 0.6 and d/R = 0.78 systems and eigen vertical displacement field distributions. From bottom to top, the eigen vertical vibration modes at center and edge of BZ are presented. Corresponding symmetrical symbols and Zak phase of each band are marked in the band structure.

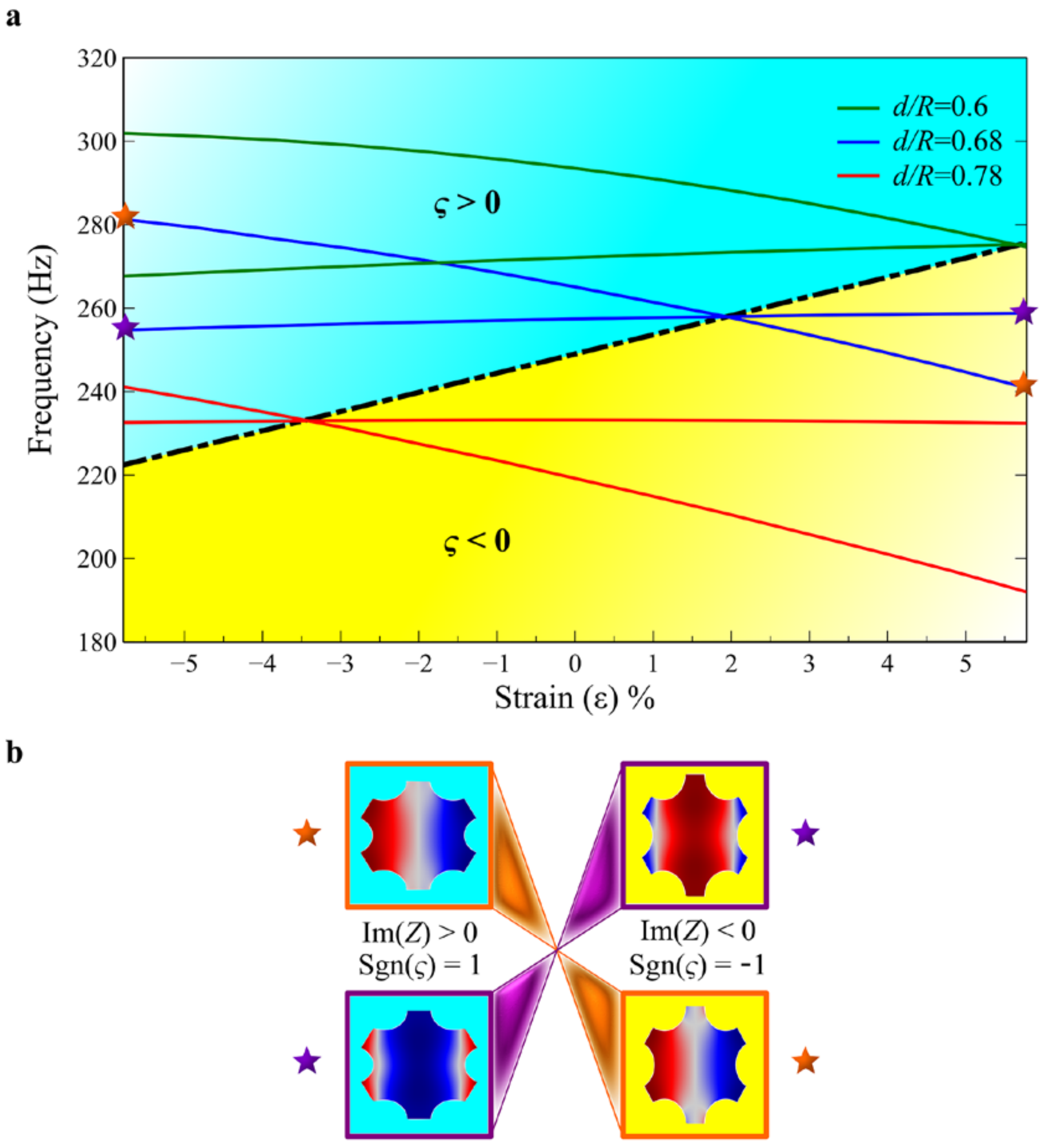

Supplementary Figure 3 | Topological phase diagram under vertical deformation. a, Topological phase diagram as a general design scheme consists of two domains separated by topological transition line (black dash-dotted line) that is formed by connecting the topological transition points of different filling ratio systems. Green, blue and red lines indicate three different filling ratio systems (d/R = 0.6, 0.68, 0.78). The opposite band gap signs ς are shown in the yellow and cyan regions. **b**, Take d/R = 0.68 as an example. Left panel shows the real parts of eigen vertical vibration modes under strain $\varepsilon = -5.77\%$ and right panel shows them at $\varepsilon = 5.77\%$, which clearly suggests band inversion. Corresponding stars are beside the vibration modes referred in **a**. The imaginary part of surface impedance and band gap sign are marked between two vibration modes. All data are from numerical simulation.

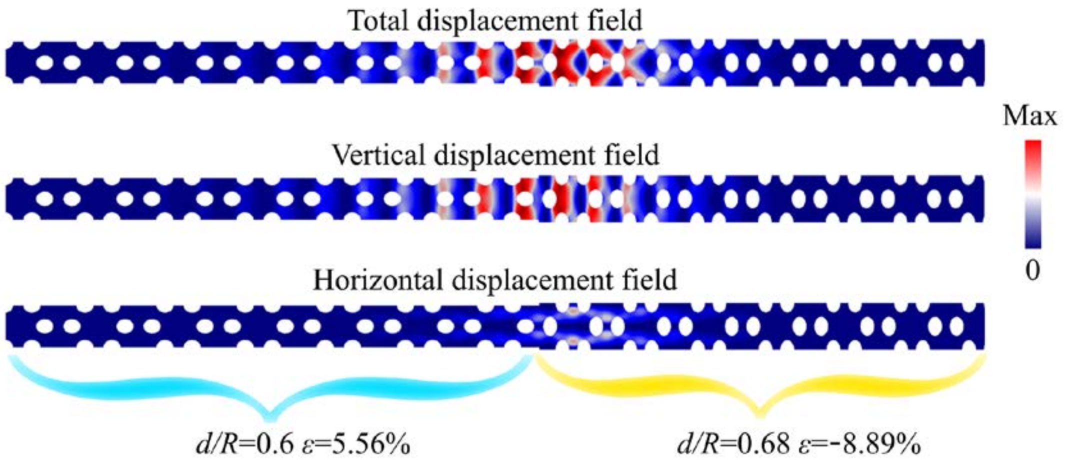

Supplementary Figure 4 | The eigen mode of the topological flat band. From top to bottom, the total displacement field distribution and two sub-displacement field distributions are shown, indicating topological interface state of transversal polarization.

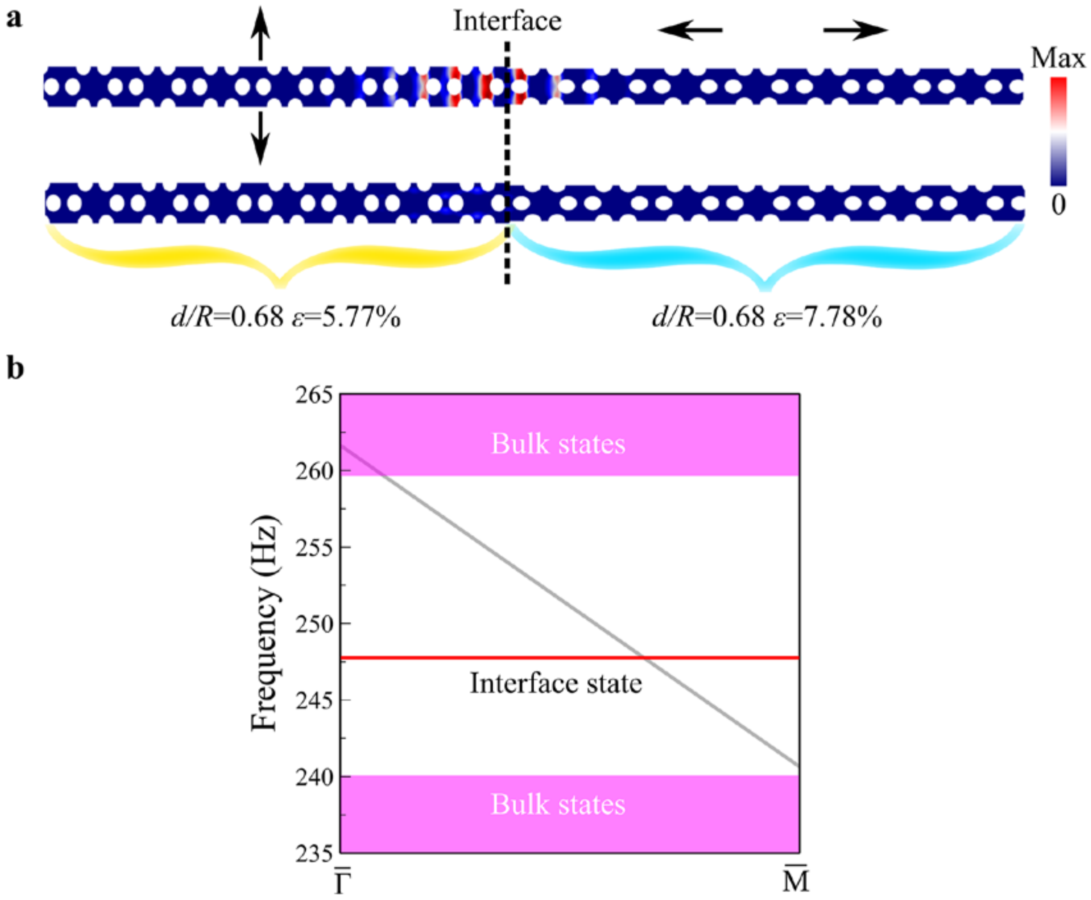

Supplementary Figure 5 | Numerical observation of topological interface state in same filling ratio system. a, Supercell used to calculate projected band structure. The origin ribbon is formed by 30 unit cells with d/R = 0.68. The left part is under vertical tensile strain ε = 5.77% and the right part is under horizontal tensile strain ε = 7.78%. The first panel is vertical displacement field and the second panel is horizontal displacement field. **b**, The simulated projected band structure

along $\overline{\Gamma}\overline{M}$ direction with transverse interface mode. Red line indicates interface state independent from bulk states (magenta region) and gray lines indicates longitudinal wave mode.

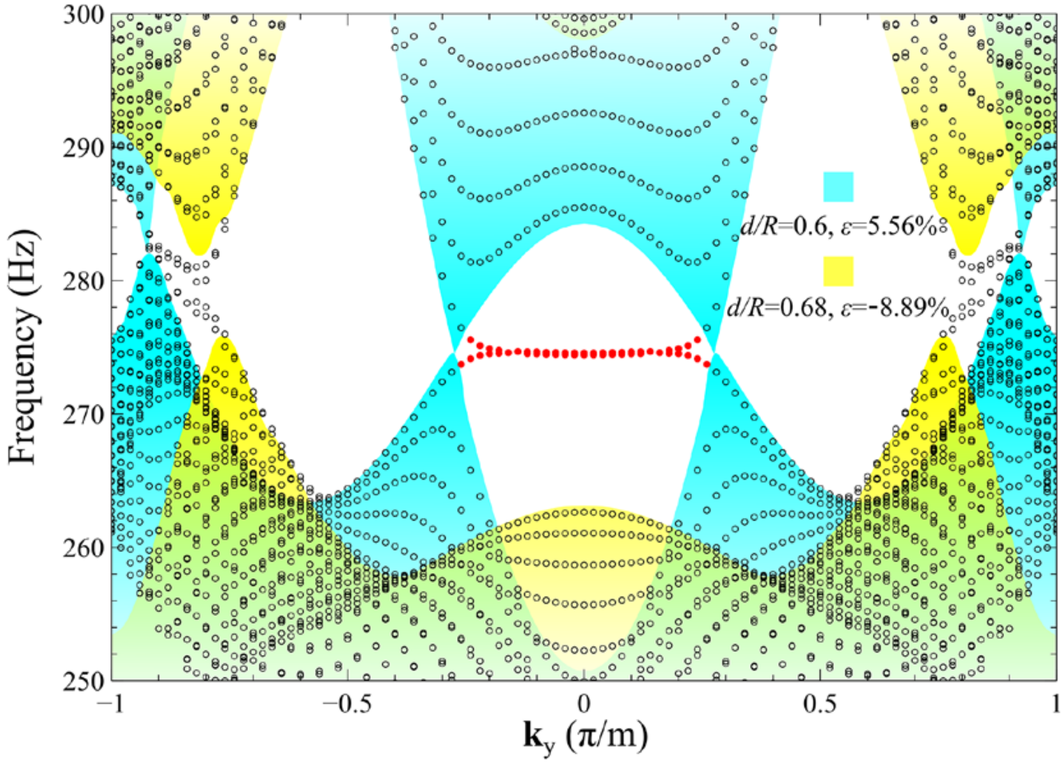

Supplementary Figure 6 | Projected band structure along $\mathbf{k}_y$ direction. Cyan region indicates the projected band structure with filling ratio of d/R = 0.6 and tensile strain ε = 5.56%. Yellow region shows the projected band structure with filling ratio of d/R= 0.68 and compressive strain ε = −8.89%. Dotted lines show the projected band structure of two edge-to-edge joint metamaterials. A topological flat band is shown near $\mathbf{k}_y$ = 0 marked in red dots.

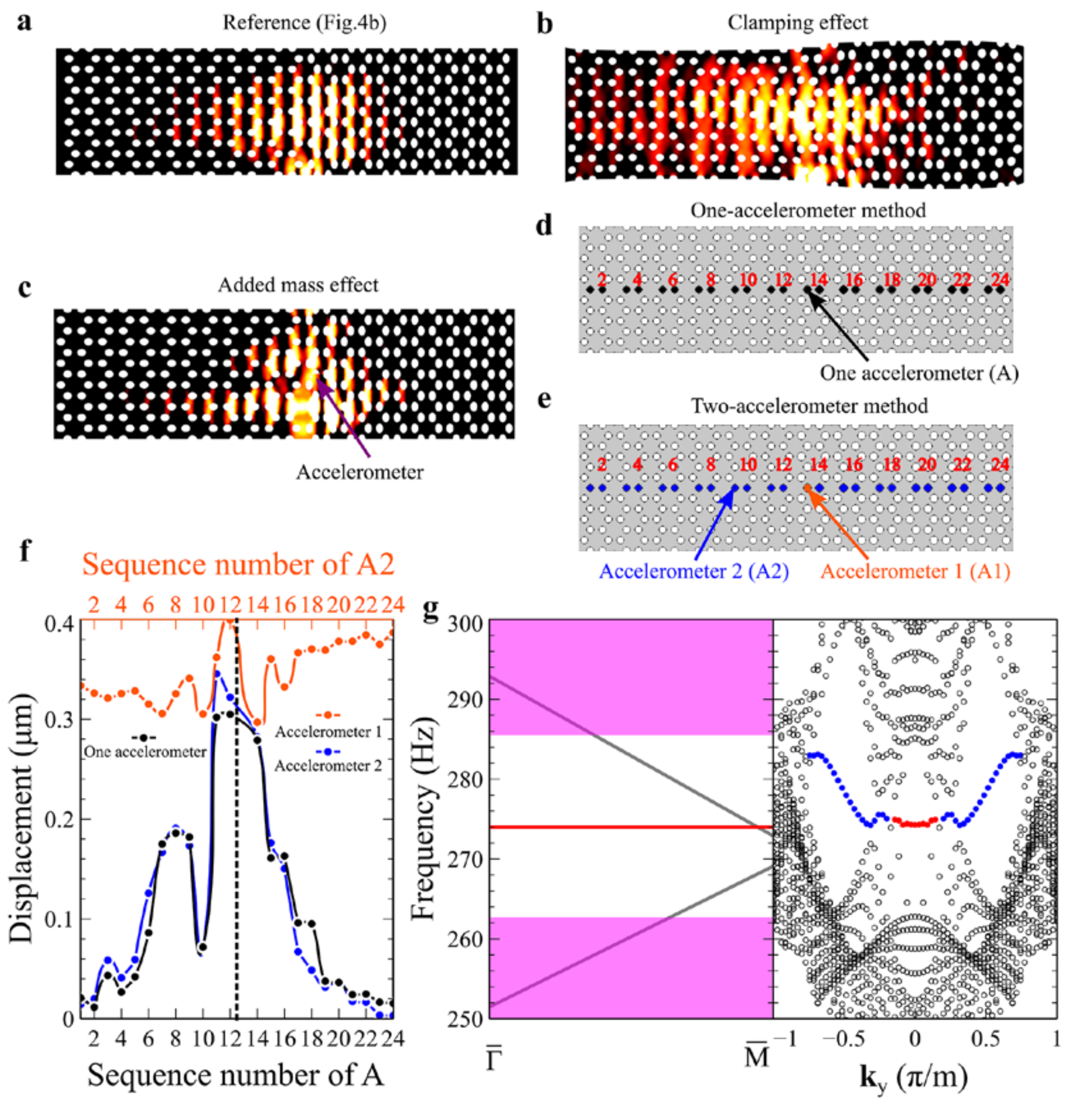

Supplementary Figure 7 | Clamping effect and accelerometer effect. a, The simulated topological interface state of (0.6, 5.56%|0.68, −8.89%) metamaterial shown in Fig. 4b. **b**, The simulated topological interface state of (0.6, 5.56%|0.68, −8.89%) metamaterial with an intermediate region modeled by deformed geometry. **c**, The simulated topological interface state of (0.6, 5.56%|0.68, −8.89%) metamaterial with an accelerometer in the number 13 hole. **d**, The schematic of one-accelerometer method to measure the topological interface state. One accelerometer is used to measure the displacement field by detecting the displacement from number 1 hole to number 24 hole. **e**, The schematic of two-accelerometer method to measure the topological interface state and to confirm the tiny effect of the added mass. One accelerometer (A2) is inserted into the holes from number 1 to number 24 to measure the displacement and the other one (A1) is inserted into the number 13 hole as an obstacle. The data from the "obstacle" accelerometer is also recorded. **f**, The comparison between measurements using one-accelerometer method and two-accelerometer method. The black curve shows the experimental results using one accelerometer. The blue curve shows the experimental results when A1 is placed at the number 13 hole and A2 is used to measure the displacement. Note that the data of number 13 hole is not measured because of the occupation of number 13 hole by A1. The orange curve shows the variation of vibration in interface position when A2 changes the measured hole. **g**, The left panel

shows the simulated projected band structure along $\mathbf{k}_x$ direction indicated by $\overline{\Gamma}\overline{\mathrm{M}}$ with an accelerometer in the number 13 hole. The bars above Γ and M are used to distinguish the Γ and M from band structure of unit cell. Red line indicates interface state independent of bulk state (magenta region) and gray lines indicate longitudinal wave modes. The right panel shows the simulated projected band structure along $\mathbf{k}_y$ direction with an accelerometer in the number 13 hole. A topological band is shown near $\mathbf{k}_y = 0$ marked in red dots. The blue dots show the longitudinal modes arisen from the added mass.

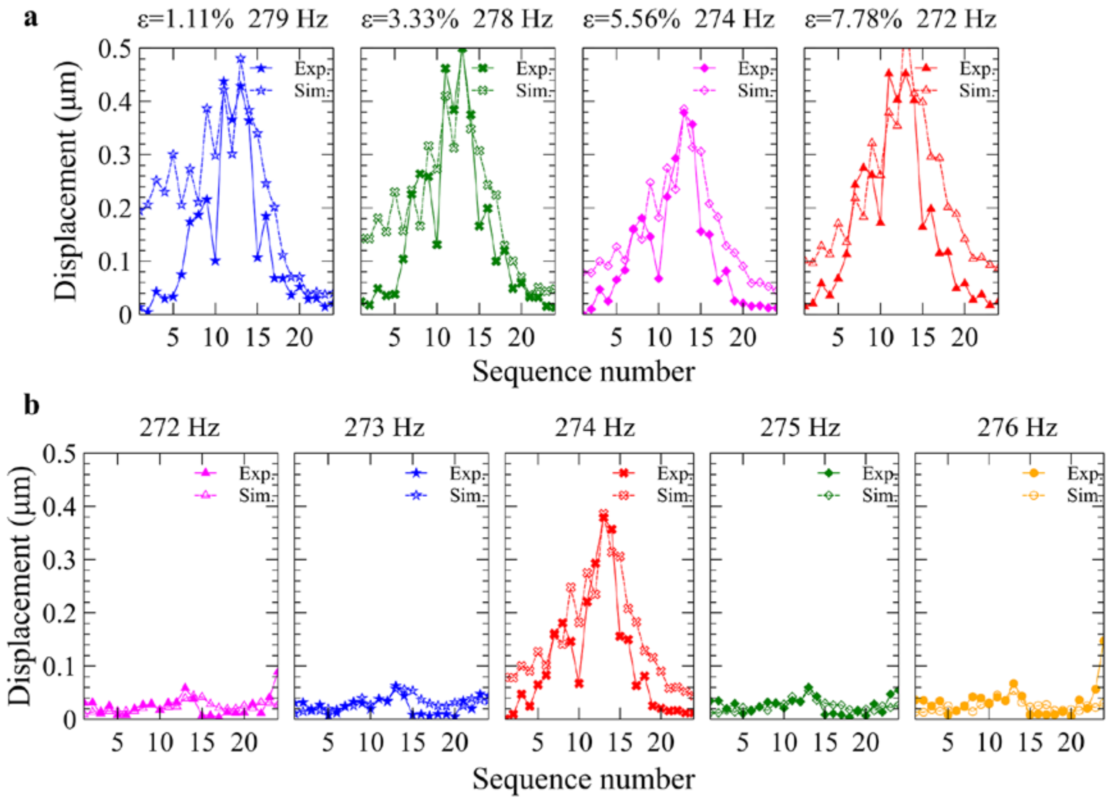

Supplementary Figure 8 | The comparison of experiment results and simulation results. a, From left to right, the experimentally measured and numerically simulated displacement field distributions at four selected strains and corresponding frequencies. The markers and colors have their correspondences in Fig. 5b. **b**, From left to right, the experimentally measured and numerical simulated displacement field distributions at five selected frequencies at strain of 5.56% along the purple dashed line in Fig. 5b, 272 Hz, 273 Hz, 274 Hz, 275 Hz and 276 Hz.

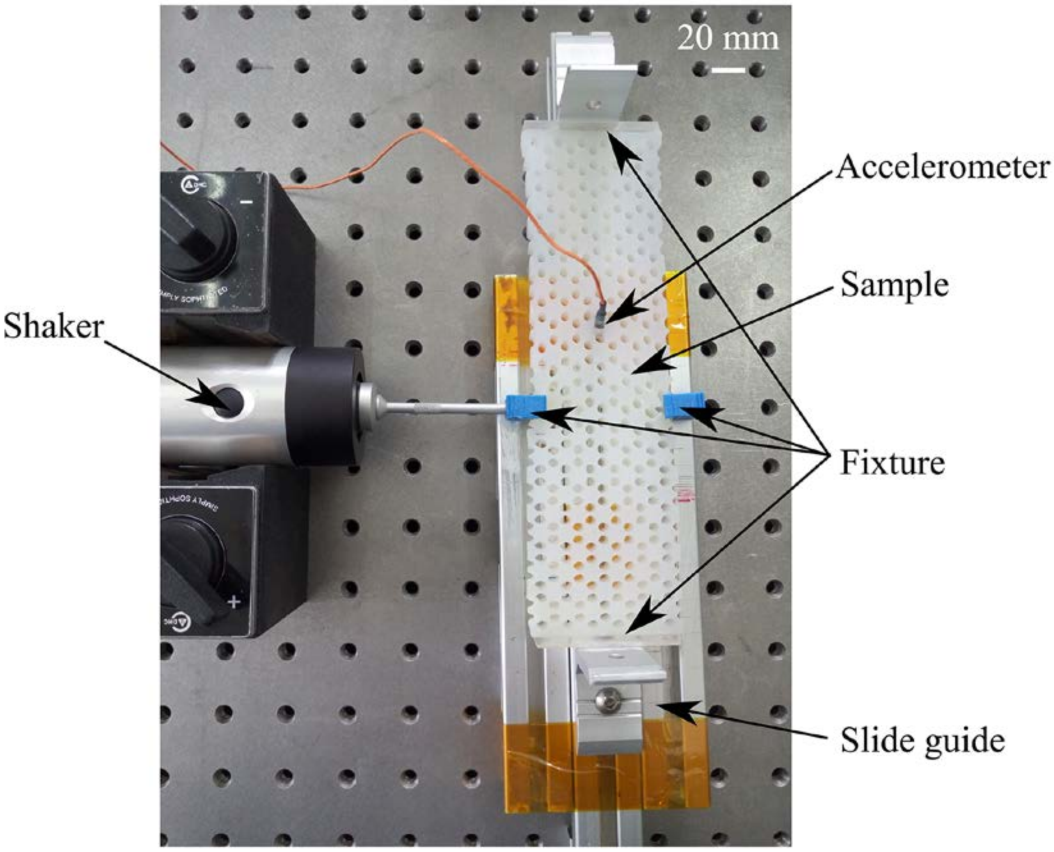

Supplementary Figure 9 | Experimental setup showing sample, shaker, accelerometer, fixtures and slide guide. Fixture on one side has a hole in order to let the rigid vibration rod touch the sample. In addition, fixtures are glued to two edges of the interface and the slide guide by using cyanoacrylate adhesive. On both sides of the sample, acrylic plates are glued to the sample to make applied strain uniform.

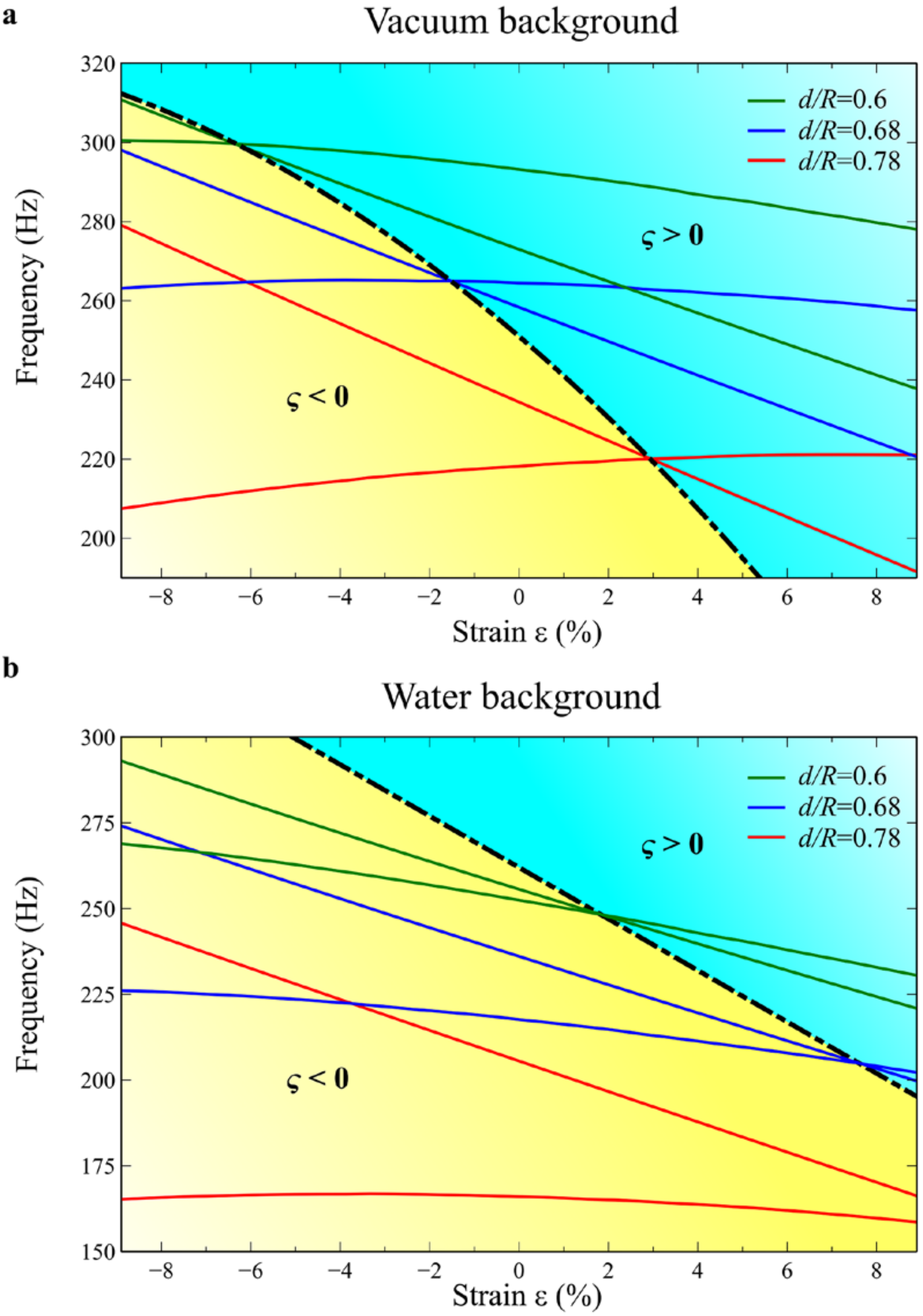

Supplementary Figure 10 | Topological phase diagram with vacuum and water background respectively. a-b, Vacuum background in **a** and water background in **b**. Green, blue and red lines indicate three different filling ratio systems (d/R = 0.6, 0.68, 0.78). The opposite band gap signs of cyan and yellow regions are $\varsigma > 0$ and $\varsigma < 0$.

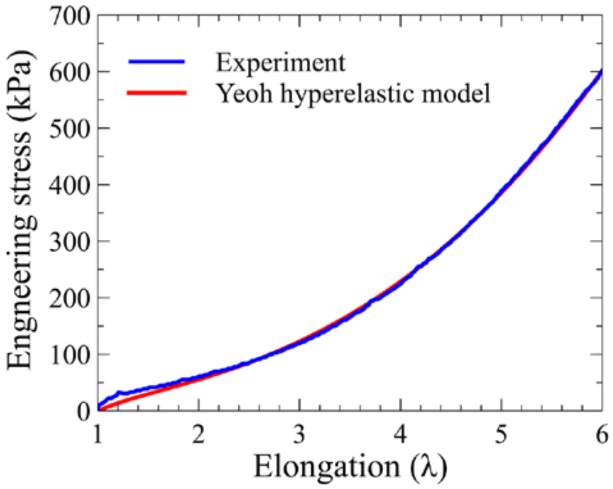

Supplementary Figure 11 | Mechanical test for Ecoflex 0030 under ASTM D412 standard. Blue curve is obtained by tensile experiment and fit to Yeoh hyperelastic model (red curve).

Supplementary Note 1 | Calculation of the Zak phase of bulk band

The Zak phase is a Berry phase across an appropriately chosen one-dimensional Brillouin zone. Because the unit cell of our system has spatial inversion symmetry, we use the symmetry analysis method to calculate the Zak phase of bulk band, which is more convenient than numerical calculation[1,2]. If we only consider a certain direction with a fixed $\mathbf{k}_y$, the system is actually reduced to be a one-dimensional problem. For each bulk band, the Zak phase should be π if the eigenmodes at center and edge of Brillouin zone possess different symmetries. Otherwise, the Zak phase is 0. The high symmetry points we choose are P point ($\mathbf{k}_x$ = 0, $\mathbf{k}_y = \mathbf{k}_{||}$) and Q point ($\mathbf{k}_x = \pi/|a_2|$, $\mathbf{k}_y = \mathbf{k}_{||}$) shown in Supplementary Fig. 2a. Note that the vertical coordinate of K point (the corner of BZ) is y_k and then $\mathbf{k}_{||}$ equals to $0.2\times y_k$. Since the systems at P point and Q point possess σ_x mirror symmetry, the eigenmodes should be either oven or odd function with respect to the center of unit cell[3]. Supplementary Fig. 2b and Supplementary Fig. 2c present the transverse wave band structure of d/R = 0.6 and d/R = 0.78 respectively. When we check the real part of eigen vertical displacement field distributions, according to the criterion, the Zak phase of the first and the second band of d/R = 0.6 system is π and 0. Similarly, the Zak phase of the first and the second band of d/R = 0.78 system is 0 and π. Further, we can obtain the sign of band gap associated with Zak phase by a simple expression[4]:

$$\mathrm{sgn}\left(\varsigma^{(n)}\right) = (-1)^n(-1)^l e^{i\sum_{m=0}^{n-1}\theta_m^{Zak}} \quad (1)$$

Where n is the sequence of band gap and l is the number of crossing points beneath this band gap.

Supplementary Note 2 | Measurements of topological interface states

We carry out static and dynamical measurement of topological interface states. First, we use two different types of clip to fix the sample. One is used to stretch/compress/fix the sample and placed at both sides of the sample (transparent acrylic plate in Supplementary Fig. 9), and the other is glued at two edges of the interface to realize independent motion of each soft metamaterial (blue 3D printing PLA in Supplementary Fig. 9).

Note that the two blue clips in Supplementary Fig. 8 could generate a small strain intermediate region, as shown in Supplementary Fig. 7b. We numerically simulated the clamping situation by setting a boundary constraint to the corresponding position where the clamps locate and a harmonic force at the edge of the interface. The simulation model with the actual geometry after mechanical deformation was used. As presented in Supplementary Fig. 7b, the resultant simulation results show that the transverse modes are located in the interface region, which is consistent with the simulation results in Fig. 4b and Supplementary Fig. 7a. Thus, the clamping has no significant effect on the generation of the topological states.

The transverse wave is generated by a shaker and the vibration is transmitted by a rigid rod. We measure the interface state characterized by displacement field distribution using accelerometer at several levels of applied deformation. At the strain level of interest, we immobilize the specimen by fixing the slide block on the slide guide and measure the displacement in a set of number labeled air holes marked in Fig. 4b. In order to measure different propagation directions of elastic wave in two parts of metamaterials, we choose two holes on each side (colored in magenta and blue) according to simulation results and put the accelerometer into them. The exciter provides a random vibration (250 Hz~320 Hz) and the spectra is obtained by Fourier transforms. The ambient noise is also recorded when the sample is statically placed and is below 0.5 nm at the frequency range of interest, which does not affect our results.

As for dynamical measurement, the basic experimental setup is the same as static measurement. We compress the d/R = 0.68 soft metamaterial to strain ε = –8.89% and fix it. The accelerometer is put into the hole at interface (Number 12). The other part d/R = 0.6 is stretched and compressed repeatedly while the vibration (274 Hz) is generated by the shaker constantly.

When we deform the metamaterial, the measured acceleration value is fluctuating. The appearance of the topological interface states is observed when the acceleration value reaches the maximum, which is marked in the Supplementary Movie. The topological interface states have the typically enhanced localized field, so the dynamical curve reflects the appearance and disappearance of interface state. Due to the nature of elastomer, this process can be repeated thousands of times and the feature of strong localization of field remain stable.

In order to investigate whether the measurement method affects experimental results, we carry out another experiment: one accelerometer is inserted into the holes to measure the displacement and the other one is inserted into the number 13 hole as an obstacle. The data from the “obstacle” accelerometer are also recorded. The measured displacement field (blue curve in Supplementary Fig. 7f) is almost the same as the former one (black curve in Supplementary Fig. 7f). Besides, the displacement at the number 13 hole is nearly stable when the accelerometer moves from number 1 to number 24 hole (orange curve in Supplementary Fig. 7f). The additional experiment suggests our technique can hardly affect the results.

Supplementary Note 3 | Governing equations and wave propagation analysis

We delineate the undeformed and the deformed states by Ω_0 and Ω, and the material and spatial points by X and x, respectively. The motion of the material is shown as affine mapping that assigns material points X to spatial points x[5].

$$x_i = \mathcal{T}_i(X_I, t) \quad (2)$$

The deformation gradient of the motion is defined as:

$$F_{iJ} = \frac{\partial x_i}{\partial X_J} \quad (3)$$

In our case, the local term of linear momentum balance can be expressed in the undeformed configuration as[6]:

$$\frac{\partial P_{iJ}}{\partial X_J} - \rho \frac{D^2 U_i}{D t^2} = 0 \quad (4)$$

where **P** is the first Piola-Kirchhoff stress, **U**=**x**-**X** is the displacement field. In our case, the hyperelastic model is described by the strain energy density function *W*. So the first Piola-Kirchhoff stress **P** can be written as:

$$P_{iJ} = \frac{\partial W}{\partial F_{iJ}} \quad (5)$$

Further, the Cauthy stress can be expressed as $\boldsymbol{\sigma} = J^{-1}\boldsymbol{P}\boldsymbol{F}^{T}$, where $\mathrm{J} = \det(\mathbf{F})$. Therefore, the equation (4) can be described spatially:

$$\frac{\partial \sigma_{ij}}{\partial x_j} - \rho \frac{\partial^2 u_i}{\partial t^2} = 0 \quad (6)$$

Then, we consider a small perturbation superimposed on the given deformed configuration that takes the continuous material to a new equilibrium. The incremental problem can be described in the deformed configuration:

$$\frac{\partial \overset{\frown}{\sigma}_{ij}}{\partial x_j} - \rho \frac{\partial^2 \overset{\frown}{u}_i}{\partial t^2} = 0 \quad (7)$$

Where the bracket symbol on the quantity denotes the increment of the corresponding quantity. After employing push-forward transformations based on linear momentum[7], we obtain:

$$\overset{\frown}{\sigma}_{ij} = J^{-1}\,\overset{\frown}{P}_{iJ}\,F_{jJ} = C_{ijkl}\frac{\partial \overset{\frown}{u}_k}{\partial x_l} \quad (8)$$

Where $C_{ijkl} = J^{-1}F_{jJ}F_{lL}\frac{\partial^2 W}{\partial F_{iJ}\partial F_{kK}}$ (9) represents the spatial elasticity tensor.

Since the amplitude of elastic wave is small, the propagation can be described by

$$\overset{\frown}{\boldsymbol{u}}\,(\boldsymbol{x}, t) = \boldsymbol{u}(\boldsymbol{x})e^{-i\omega t} \quad (10)$$

$$\overset{\frown}{\boldsymbol{\sigma}}\,(\boldsymbol{x}, t) = \boldsymbol{\sigma}(\boldsymbol{x})e^{-i\omega t} \quad (11)$$

So that equation (7) becomes

$$\nabla \cdot \boldsymbol{\sigma} + \rho\omega^2\boldsymbol{u} = 0 \quad (12)$$

Since our elastic metamaterial is a periodic structure characterized by a unit cell, any periodic function satisfies the condition:

$$\phi(\mathbf{x} + \mathbf{r}) = \phi(\mathbf{x}) \quad (13)$$

where $\mathbf{r} = r_1\boldsymbol{a}_1 + r_2\boldsymbol{a}_2$ (14)

where r_1 and r_2 are arbitrary integers. In deformed configuration, the basic vectors $\boldsymbol{a}_1$ and $\boldsymbol{a}_2$ are different from the undeformed ones due to the change of reciprocal lattice.

The reciprocal lattice vectors $\boldsymbol{b}_1$ and $\boldsymbol{b}_2$ can be derived from:

$$\boldsymbol{a}_i \cdot \boldsymbol{b}_j = 2\pi\delta_{ij} \quad (15)$$

Specifically, in 2D lattice:

$$\boldsymbol{b}_1 = 2\pi\frac{\boldsymbol{a}_2 \times \boldsymbol{e}_z}{\boldsymbol{a}_1 \cdot (\boldsymbol{a}_2 \times \boldsymbol{e}_z)} \quad (16)$$

$$\boldsymbol{b}_2 = 2\pi \frac{\boldsymbol{e}_z \times \boldsymbol{a}_1}{\boldsymbol{a}_1 \cdot (\boldsymbol{a}_2 \times \boldsymbol{e}_z)} \quad (17)$$

Thus, the reciprocal lattice can be described by:

$$\mathbf{g} = g_1 \boldsymbol{b}_1 + g_2 \boldsymbol{b}_2 \quad (18)$$

Any function $\varphi(\mathbf{k})$ in reciprocal space satisfies[8]:

$$\varphi(\mathbf{k} + \mathbf{g}) = \varphi(\mathbf{k}) \quad (19)$$

In order to obtain band structures for different strain levels, Bloch boundary condition is applied to the deformed lattice:

$$\mathbf{u}(\mathbf{x} + \mathbf{r}) = \mathbf{u}(\mathbf{x}) e^{i\boldsymbol{k}\cdot\boldsymbol{r}} \quad (20)$$

Then we solve the equation (12) in the states for different strain levels. Due to the symmetry in equation (19), we focus on the first Brillouin zone. We calculate the band structure along the path Γ-M-K-Γ shown in Fig. 1c and Fig. 1d.

Supplementary References:

1. Kohn, W., Analytic Properties of Bloch Waves and Wannier Functions. *Phys. Rev.* **115,** 809-821 (1959).
2. Zak, J., Symmetry criterion for surface states in solids. *Phys. Rev. B* **32,** 2218-2226 (1985).
3. Huang, X., Xiao, M., Zhang, Z. & Chan, C. T., Sufficient condition for the existence of interface states in some two-dimensional photonic crystals. *Phys. Rev. B* **90,** 075423 (2014).
4. Xiao, M., Zhang, Z. Q. & Chan, C. T., Surface Impedance and Bulk Band Geometric Phases in One-Dimensional Systems. *Phys. Rev. X* **4,** 021017 (2014).
5. Zhang, P., Parnell, W. J., Soft phononic crystals with deformation-independent band gaps. *Proc. R. Soc. A* **473,** 2200 (2017).
6. Holzapfel, G. A., Nonlinear solid mechanics: A continuum approach for engineering. *Wiley, Chichester* (2000).
7. Wang, P., Shim, J. & Bertoldi, K., Effects of geometric and material nonlinearities on tunable band gaps and low-frequency directionality of phononic crystals. *Phys. Rev. B* **88,** 014304 (2013).
8. Brillouin, L. Wave Propagation in Periodic Structures. *McGraw-Hill* (1946).
