# Supplementary material for: Observation of elastic topological states in soft materials
Source: Nat Commun. 2018 Apr 10;9:1370. doi: 10.1038/s41467-018-03830-8 (PMC5893582; doi:10.1038/s41467-018-03830-8)
Supplement: Supplementary file 3 — Description of Additional Supplementary Files [file 41467_2018_3830_MOESM3_ESM.pdf]

Description of Additional Supplementary Files

File Name: Supplementary Movie 1
Description: The dynamical manipulation of topological interface states by mechanical deformation. The movie shows a time dependent acceleration signal when the elastic metamaterial is stretched or compressed. The appearance of the topological interface states is observed when the acceleration value reaches the maximum.
